# Research Note: A deep learning method segments chicken keel bones from whole-body X-ray images

**DOI:** 10.1016/j.psj.2024.104214

**Published:** 2024-08-13

**Authors:** Moh Sallam, Samuel Coulbourn Flores, Dirk Jan de Koning, Martin Johnsson

**Affiliations:** ⁎ Department of Animal Biosciences, Swedish University of Agricultural Sciences, Box 7023, 750 07, Uppsala, Sweden; † Department of Biochemistry and Biophysics, Stockholm University, Tomtebodavägen 23A, 171 65, Solna, Sweden

**Keywords:** keel bone, sternum, machine deep learning, segmentation, laying hen

## Abstract

Most commercial laying hens suffer from sternum (keel) bone damage including deviations and fractures. X-raying hens, followed by segmenting and assessing the keel bone, is a key to automating the monitoring of keel bone condition. The aim of the current work is to train a deep learning model to segment the keel bone out of whole-body x-ray images. We obtained full-body x-ray images of laying hens (n = 1,051) and manually drew the outline of the keel bone on each image. Using the annotated images, a U-net model was then trained to segment the keel bone. The proposed model was evaluated using 5-fold cross validation. We obtained high segmentation accuracy (Dice coefficients of 0.88–0.90) repeatably over several validation folds. In conclusion, automatic segmentation of the keel bone from full-body x-ray images is possible with good accuracy. Segmentation is a requirement for automated measurements of keel geometry and density, which can subsequently be connected to susceptibility to keel deviations and fractures.

## INTRODUCTION

Poultry is a global, high-volume, high-throughput, low-margin industry. One consequence of this is that commercial poultry (layers and broilers) are heavily genetically optimized. Bone fractures, often featuring keel bone, are present in the majority (up to 80%) of commercial laying hens (Thøfner et al., 2021), causing a significant welfare issue (Nasr et al., 2012) and drop in egg production (Wei et al., 2020). Bone traits tend to be heritable (Bishop et al., 2000), the industry therefore desires genetic improvements which would lead to reduced bone fractures. This leads to the prerequisite question for genetic improvements – how can the industry monitor thousands of birds for bone conditions?

The large-scale x-raying of live birds on-farm has been considered as a potential solution by the poultry breeding community (e.g., Rufener et al., 2018; Jung et al., 2022). However, the existing postimaging methods require a human operator to indicate key points on chicken bone x-ray images, and from these compute a fracture propensity (Wilson et al., 2022), or keel bone geometry (unpublished work). The need for a skilled operator to manually provide these annotations makes the method time consuming, prone to noise, and impractical given the number of birds involved in poultry facilities. Automating the postimaging methods is essential for successful implementation of x-ray imaging as a novel phenotype in selective breeding.

Image segmentation and classification are 2 computer vision processes that can be automated using machine learning approaches. Segmentation refers to partitioning an image into 2 or more regions – in this case keel bone vs. the background including other bones. To be technical, each pixel is labeled as keel bone vs. background. Classification means labeling the entire image as belonging to 1 of 2 or more classes. In keel bone case, 2 classes can be zero fractures vs. 1 or more fractures. If we desire 4 classes, these could be, for example, 0, 1, 2, 3 and more than 3 fractures. Regression can also be an alternative to the multiclass classification, with the difference that the output will be on a continuous rather than a discrete scale.

Keel bone damage has been considered one of the major welfare concerns in laying hens while the tibia bone has often been used to measure bone strength in a consistent manner. For this reason, these bones are of particular interest. We propose that the first step to assess bone quality from x-ray images will be to segment these bones. The focus of the current work is therefore the segmentation step, specifically keel bone segmentation.

To automate keel bone segmentation from the whole-body image, a model is trained to distinguish the keel pixels from the nonkeel pixels. Technically this requires whole-body images and annotation where keel pixels are given a white color (numerically 1) while nonkeel pixels are black (numerically 0). A deep learning model is then trained to extract the features of the images as numerical values and estimate the weights of these features that can predict the annotation, i.e., predicting which pixels are keel pixels. The obtained predictive model is then evaluated on images that were never used in the training, and if it accurately segments the keel, the model can be used for automatic segmentation of further images.

**Figure 1:**
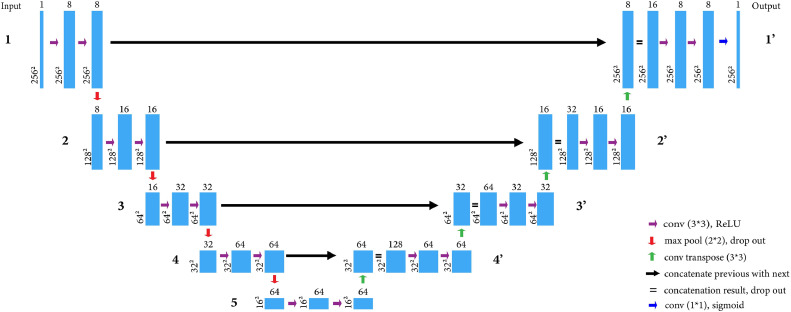
U-net architecture in our implementation. Blue columns represent U-net layers, with dimensions on the left and number of channels on top. Max pooling (red arrows) down samples layers by 2× in each dimension to extract key features in blocks 1 to 5. Convolution (purple arrows) is used to create additional layers with the same dimension but sometimes different number of channels. Convolution transpose (green arrows) expands the layers in blocks 4′ to 1′. Spatial information is recovered through the skip connections (black arrows). The final convolution (blue arrow) compresses 8 channels into 1.

In this study, we use U-net, a widely used convolution neural network to enable the machine to learn image segmentation (Ronneberger et al., 2015). The first half of U-net is a contracting path, where the resolution of images is progressively reduced in successive layers (blue bars, Figure 1), to increase the abstraction, thus extracting the key features. The “U” in the name refers to the way in which the layers then use deconvolution or upsampling to recover spatial features in an expansive path. The U-net model also has skip connections, which concatenate the contracting and expanding parts. This means the extracted key features are combined with their spatial features, enabling the machine to learn not only the object of interest but also its location. In this paper, we aimed to use U-net to automate keel segmentation from whole-body x-ray images which will facilitate large-scale phenotyping of the keel bone.

## MATERIALS AND METHODS

### Birds

Images of Bovans Brown hybrids were generated with a portable x-ray machine (Medivet Scandinavian AB, Ängelholm, Sweden). The x-ray exposure setting was 65 kV and 1.0 mAs with 1-meter distance between the x-ray tube and the flat panel detector. The methods of data collection are described in Sallam et al. (in review). The study was conducted in accordance with the local legislation and institutional requirements with approval from the Gothenburg Local Ethics Committee of the Swedish National Board for Laboratory Animals (Reference 5.8.18-16645/2020).

### Gold Standard Mask

To create the Gold Standard mask (**GSM**), Sallam (a veterinarian) hand-traced the outline of the keel bone over each whole-body x-ray image using a Wacom Cintiq pen display with GNU Image Manipulation Program GIMP (www.gimp.org). The outline was then filled in with white and the background filled in with black, in an automated step coded using the open-source computer vision python package (www.opencv.org). The whole-body x-ray images and GSM are available at https://doi.org/10.5281/zenodo.11172093.

### U-Net

The architecture of U-net can be adjusted for a given implementation. In the current work, the input layer has a resolution of 256 × 256 with a channel depth of 1. Succeeding blocks decrease resolution by factors of 2, until the bottom layer has resolution of 16 × 16 with 64 filters. Other differences in resolution, number of layers, channel depth with respect to (Ronneberger et al., 2015), are given in Figure 1. The U-net was coded using the TensorFlow python library (Abadi et al., 2016) and available from the GitHub repository: https://github.com/sallamslu/Keel-bone-segmentation.

### Cross-Validation Design and Evaluation Metrics

A total of 1051 x-ray images and their respective GSM were split into 80% for training, 12% for validation, and 8% for testing. The splitting was randomized and repeated 5 times to ensure the 5-fold cross-validation. The training is an iterative process, initiated by giving an arbitrary weight to each pixel value on the images. The pixels’ weights are then updated, along the iteration epochs, to minimize the difference between the network output (predicted mask, real number ranging 0–1) and the GSM (0 for the nonkeel pixels, or 1 for the keel pixels). This difference is referred to as the loss or error function, in this case computed with the cross-entropy function. The accuracy was reported using the Dice coefficient, which relates the overlap of predicted mask and GSM, to the sizes of the 2. Good convergence is indicated by loss approaching zero and accuracy approaching unity over epochs. Both metrics should be similar for test and validation sets, to indicate that overfitting has not occurred.

## RESULTS AND DISCUSSION

On x-ray images of chickens, the pixel contrast between keel bone and background is small, thus, keel bone outlines are not fully clear, and overlap sometimes with adjacent tissues. This challenges the most recent pretrained models like the Segment-Anything Model (Kirillov et al., 2023) to segment keel bone from the whole-body x-ray images. For that reason, we opted to train an all-new model based on U-net with the prerequisites to create 1051 GSM of keel bones from whole-body X-ray images. With the help of simple scripts to automate opening and exporting images in GIMP software, as well as a pen display to draw keel outlines, creating 1,051 GSM of keel bones required only 7 person-hours of manual effort in the current work.

**Figure 2:**
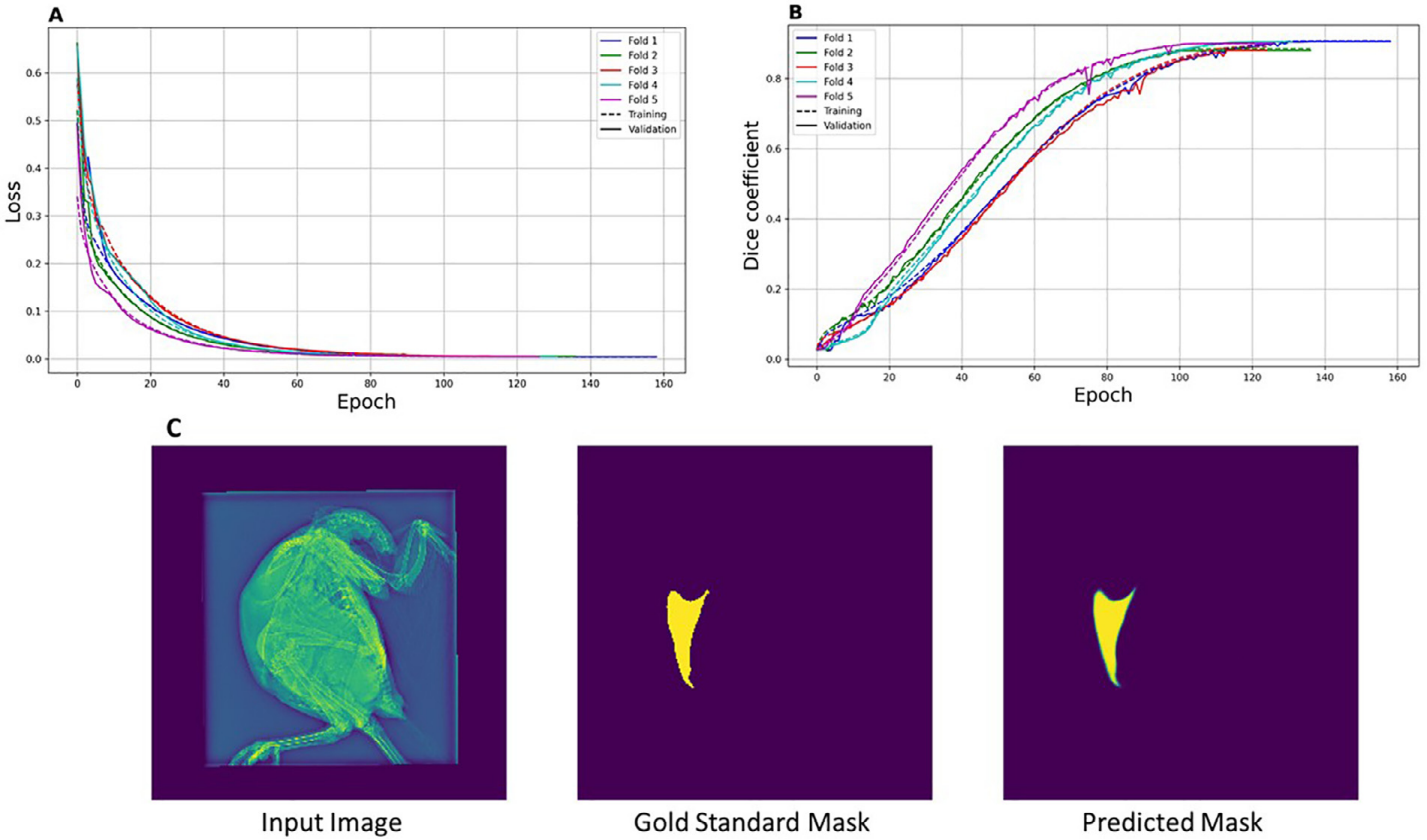
Loss curves (A) and Dice coefficient (B) for the 5 folds of training and validation. For each fold, a different test-train split was used. Model performance example (C): Left: whole-body X-ray image. Center: gold standard mask. Right: predicted mask using our converged model.

Our U-net model converged well, loss and Dice coefficient approach 0 and 1, respectively (Figures 2 A and 2B). The Dice coefficient, also known as F1, is a quality metric that considers false negatives (keel pixels on GSM but predicted as nonkeel), false positives (nonkeel pixels on GSM but predicted as keel), and the size of the GSM and predicted masks. Loss and Dice coefficient also had similar behavior for training and validation and across folds, thus overfitting is not suspected (Figure 2 A-B).

Test images, which the model had never seen, were in total 420 (84 in each of the 5 folds) and had their keel bones segmented with an accuracy of 0.89 averaged over the 5 folds (range: 0.88–0.90), with an example on Figure 2C. Achieving ∼0.90 segmentation accuracy with training on ∼1,000 annotated images, is quite promising. If we assumed similar setting in poultry breeding companies and the current work, automating keel bone segmentation for large numbers of images should not require extensive manual annotation efforts.

The current work does not provide an end-to-end solution for assessing chickens' keel bone for breeding purposes. Instead, it provides a dataset of annotated chicken skeleton images for further methods development and structural studies, as well as the keel bone segmentation technology, which will enable further methods to classify or quantify keel fracture occurrence. Such a classifier or quantifier could emit predictions quickly for assessment of keel bones of breeding chickens. Several dimensions will be automatically measured on the segmented keel bones and studied for their heritability as well as correlations with clinical keel bone phenotypes (e.g., fracture count and deviation size).

With a modest additional image annotation effort, the current model could also be retrained to segment bones other than keel (e.g., tibia bone), as well as other objects that are related to bone health such as eggs. Automatic segmentation of many objects on the same x-ray image (keel, tibia, and egg) would maximize the benefit of x-raying chickens.

## DISCLOSURES

The authors declare that they have no known competing financial interests or personal relationships that could have appeared to influence the work reported in this paper.
